# Molecularly-tunable nanoelectrode arrays created by harnessing intermolecular interactions†

**DOI:** 10.1039/d0sc06955h

**Published:** 2021-03-10

**Authors:** Han-Wen Cheng, Shan Wang, Marc D. Porter, Chuan-Jian Zhong

**Affiliations:** School of Chemical and Environmental Engineering, Shanghai Institute of Technology Shanghai 201418 China hwcheng@sit.edu.cn; Department of Chemistry, State University of New York at Binghamton Binghamton New York 13902 USA cjzhong@binghamton.edu; Department of Chemistry and Chemical Engineering, University of Utah Salt Lake City Utah 84112 USA marc.porter@utah.edu

## Abstract

Intermolecular interactions play a critical role in the binding strength of molecular assemblies on surfaces. The ability to harness them enables molecularly-tunable interfacial structures and properties. Herein we report the tuning of the intermolecular interactions in monolayer assemblies derived from organothiols of different structures for the creation of nanoelectrode arrays or ensembles with effective mass transport by a molecular-level perforation strategy. The homo- and hetero-intermolecular interactions can be fully controlled, which is demonstrated not only by thermodynamic analysis of the fractional coverage but also by surface infrared reflection absorption and X-ray photoelectron spectroscopic characterizations. This understanding enables controllable electrochemical perforation for the creation of ensembles or arrays of channels across the monolayer thickness with molecular and nanoscale dimensions. Redox reactions on the nanoelectrode array display molecular tunability with a radial diffusion characteristic in good agreement with theoretical simulation results. These findings have implications for designing membrane-type ion-gating, electrochemical sensing, and electrochemical energy storage devices with molecular level tunability.

## Abstract 2

Intermolecular interactions in monolayer assembly are harnessed for creating molecularly-tunable nanoelectrode arrays or ensembles.

## Graphical abstract

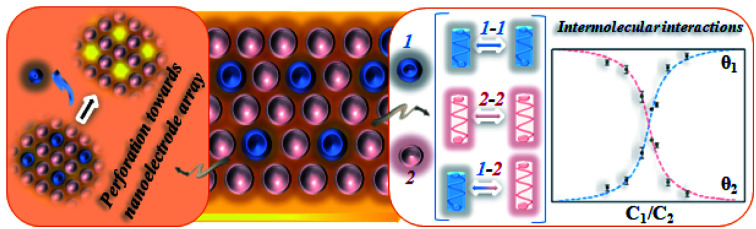

## Introduction

The fabrication of nanoelectrode ensembles and arrays has gained increasing interest in development of highly sensitive and miniaturized electrochemical devices and sensors.^1*a*^ Template synthesis of nanoporous membranes is an important pathway for the fabrication of ensembles of nanoscopic electrodes (*e.g.*, metal nanotube membranes) towards ion-permselective membrane.^1*b*^ Both bottom-up and top-down approaches have been used in the fabrication of nanopore electrode arrays or ensembles potentially viable for chip-based nano-electrochemical sensing applications.^1*c*^ The nano-confined space of nanoporous electrodes enhances electrochemical efficiency between redox probes and the electrode surface with enhanced nanoporous mass transport tunable by size, shape, and charge. The effective mass transport is also exploited for electrochemical energy conversion and storage devices,^2,3^ and materials,^2*a*^ including self-supported 1D/2D nanoarrays with different geometries for supercapacitors with high specific capacitance. Imparting nanostructure to electrodes is also desired for energy storage reactions^2*b*^ by improvement of mass transport with nanoelectrode arrays. Experimental and theoretical studies of diffusion domains based on microelectrode arrays^3^ have highlighted the importance of radial diffusion compared to microelectrode arrays and the strong dependence on the size. Major challenges for fabrication include the high-cost or complicated fabrication processes and low active-mass loading per unit area. Nanoelectrode ensembles or arrays for electrochemical applications^4^ are fabricated by both top-down electron-beam lithography or nano-imprinting for ordered arrays, and bottom-up template and self-assembly methods for random ensembles. The mass transport by radial diffusion at individual nanoelectrodes, arrays, ensembles or nanopores not only emulates transmembrane ion transport processes but also enhances the surface spatial imaging^5^ where a given footprint of a functional device could enable a larger number of active interaction points at the interface.

For the bottom-up approach to nanoelectrode fabrication by exploring the atomic and molecular structures on surfaces and interfaces, especially self-assembled systems,^6–13^ a key question is how the surface or interface can be tuned precisely for the creation of specially-structured functionality. In self-assembled monolayers (SAMs), there has been a large body of work focusing on the well-defined single-phase structural character, including earlier spectroscopic, microscopic and electrochemical studies^6^ and recent studies^7–13^ of the molecular structures of these systems. There has been limited understanding for binary or mixed SAMs due to the complexity of intermolecular interactions. *In situ* ellipsometry study of mixed monolayers showed evidence for the formation of a low refractive index region after desorption of the monolayer component.^14^ In a lateral force microscopy study of some binary monolayers formed on planar polycrystalline gold,^15^ portions of 2-aminoethanethiol were removed by selective reductive desorption and backfilled with 11-mercaptoundecanoic acid. Phase formation in mixed alkanethiol monolayers^16^ was also revealed by electrochemical quartz crystal nanobalance. For homogeneously mixed SAMs of mercaptoalkanoic acids of different chain lengths on Au(111),^17^ a pH-sensitive supramolecular switch function was revealed by breaking or restoring the hydrogen bond interaction in basic or acidic solutions. For SAMs on gold nanoparticles, recent Monte Carlo study^18^ showed that the hexanethiolate/tetradecanethiolate mixture yields Janus-like arrangement when the ligands are confined to a single nanoparticle. Atomistic simulation study^19^ showed that mixed hydrophilic and hydrophobic alkylthiols on gold nanoparticles exhibit domains of surface hydrophilicity, serving as a platform not only for structural characterizations but also for electrochemical applications. Despite the progress, the question of how the intermolecular interactions can be controlled to pin down the interfacial molecular or nanoscale functionalities remains elusive.

**Fig. 1:**
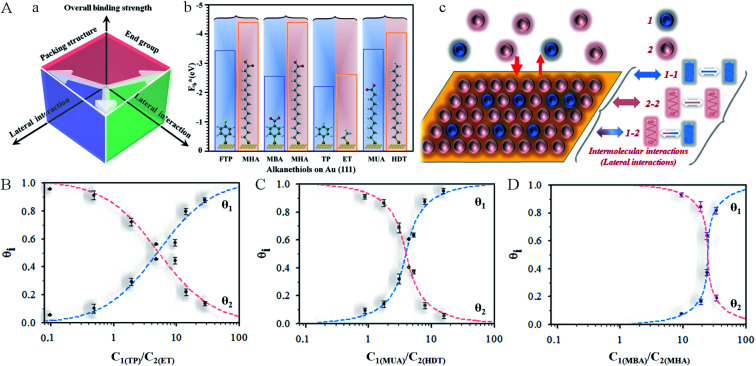
(A) (a) Illustration of the overall binding strength of monolayer assembly in terms of lateral interactions. (b) Chart showing the DFT-calculated binding strength, 
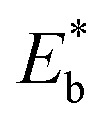
,^27,28^ for selected combinations of 4-fluorothiophenol (FTP), 16-mercaptohexdecanoic acid (MHA), mercaptobenzoic acid (MBA), thiophenol (TP), ethanethiol (ET), 11-mercaptoundecanoic acid (MUA), and hexadecanethiol (HDT) on gold (111) surface (also see Table S1†). (c) Illustration of competitive adsorption of two thiols forming a monolayer on Au(111). (B–D) Dependence of fractional coverages, *θ*_1_ and *θ*_2_, *vs.* their concentration ratio in the solution for TP (*C*_1_) and ET (*C*_2_) (B); MUA (*C*_1_) and HDT (*C*_2_) (C); and MBA (*C*_1_) and MHA (*C*_2_) (D). The dashed lines represent fitting curves based on eqn (3a) and (3b) with the fitting parameters: (B) *K*_2_/*K*_1_ = 0.7, *α*_a_ = 0.05, *α*_b_ = 2.05, and *α*_c_ = −2.0; (C) *K*_2_/*K*_1_ = 1.5, *α*_a_ = 2.8, *α*_b_ = 2.5, and *α*_c_ = 0.3; and (D) *K*_2_/*K*_1_ = 9.5, *α*_a_ = 3.8, *α*_b_ = 2.9, and *α*_c_ = 0.9.

We demonstrate here the ability to harness the intermolecular interactions and a molecular-level perforation strategy for the creation of molecularly-tunable nanoelectrode arrays or ensembles. In contrast to previous studies of mixed monolayers,^16,20–24^ the controllability of the intermolecular interactions and binding strength of the two-component monolayer to gold surface enables the molecular level tunability. Fig. 1A(a) illustrates how the overall binding strength depends on the intermolecular interactions of the two components with subtle differences in their packing structure and functional group. In other words, the overall binding strength is determined by the lateral interactions through the molecular packing structure and the functional group (end group). Fig. 1A(b) shows selected examples of the combinations to create differences in terms of the binding strength (
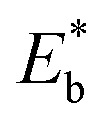
, also see Table S1†) based on density functional theory (DFT) calculations^25,26^ in several recent reports,^27,28^ which could also involve molecular packing as shown in our recent work.^25^ These differences are harnessed by tuning the alkyl chain, aromatic moiety, and functional groups in terms of hydrophobic, guest–host, protonation, hydrogen-boding, or electrostatic interactions for a wide range of sensor applications.^29–32^ The selected combination of thiolate molecules highlight the differences in molecular structure, chain length, and functional groups. It is the resulting difference in binding strength that constitutes the basis for manipulation of their intermolecular interactions and phase structures for the selective desorption by controlling the 
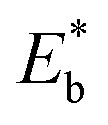
, which determines the desorption peak potential (*E*_p_).^28,33–35^ The basis for the selective reductive desorption is that *E*_p_ depends on the molecular structure, chain length, and functional groups. For example, the potential shifts negatively by ∼20 mV per (–CH_2_) group for *n*-alkanethiolate monolayers at annealed gold. By holding the applied potential (*E*_app_) at a value between the two waves corresponding to the two different components, the desorption of the short chain component is preferentially induced. This removal therefore defines the size and shape of the channels, whereas the retained long chain component functions as an impermeable framework surrounding each nanochannel, creating nanoelectrode arrays or assembles with molecular tunability.

## Results and discussion

As illustrated in Fig. 1A(a) and (b), the general design principle to tune the nanoelectrode pore size and interpore distance starts from controlling the mixing ratio of two host–guest thiol components in terms of lateral interaction and binding energy (see also Table S1†). The key is the ability to harness the intermolecular interactions and the overall binding properties. A stronger binding strength leads to a more negative desorption potential, whereas a stronger cross intermolecular interaction leads to a better mixing of the two components. These control parameters are assessed in the following subsections first in terms of the co-adsorption isotherm analysis, then the electrochemical desorption manoeuvring, and finally the nanoelectrode array evaluation.

### Co-adsorption isotherm

A novel element of our strategy is to harness the intermolecular interactions of a two-component monolayer assembly on the surface of a substrate. Consider first the equilibrium for competitive adsorption for surface sites between two thiols in a solution (sol) and the surface assembly by adsorption (ads) (**1** and **2**, Fig. 1A(c)), which is represented by eqn (1):

1 **1**_sol_ + **2**_sol_ ↔ **1**_ads_ + **2**_ads_

We further consider Frumkin adsorption isotherm,^36^ which derives from Langmuir isotherm by inclusion of attractive and repulsive intermolecular interactions between neighboring adsorbates.^37^ For the formation of a full monolayer at each mixing ratio of the two-component solutions (*θ*_1_ and *θ*_2_ are the fractional surface coverages of **1**_ads_ and **2**_ads_), eqn (2a) and (2b) can be derived,^38^2a
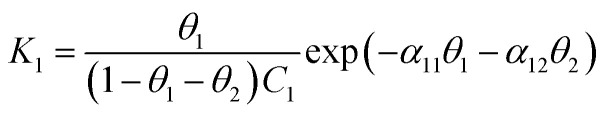
2b
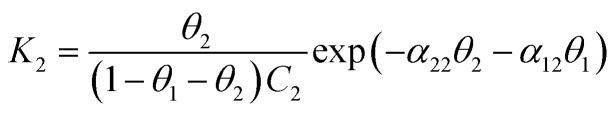
where *C*_1_ and *C*_2_ are the solution concentrations of **1**_sol_ and **2**_sol_ (at a constant total concentration), and *K*_1_ and *K*_2_ are the adsorption equilibrium constants. Eqn (2a) and (2b) introduce the intermolecular interactions between **1** and **2**, **1** and **1**, and **2** and **2**, which are described by the interaction parameter (*α*_ij_). The intermolecular interaction is attractive when *α*_ij_ > 0, and repulsive when *α*_ij_ < 0. By recasting *α*_ij_ as *α*_a_ = *α*_11_ + *α*_22_ − 2*α*_12_, *α*_b_ = *α*_22_ − *α*_12_, and *α*_c_ = *α*_11_ − *α*_12_, dividing eqn (2a) by eqn (2b), and rearranging the result, we obtain eqn (3a) and (3b) in terms of *θ*_1_ and *θ*_2_. An equation in terms of *θ*_2_ can be obtained by replacing *θ*_1_ in eqn (3a) with *θ*_2_, where *θ*_2_ = 1 − *θ*_1_.3a
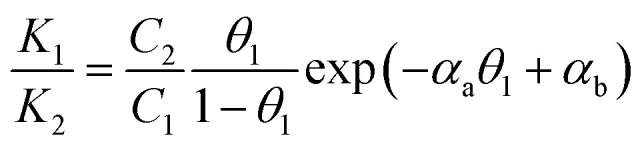
3b
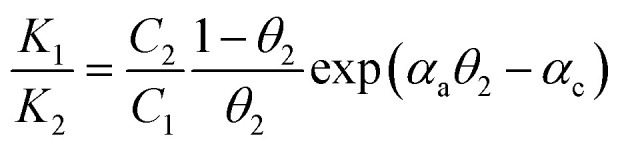


The relative homo- and hetero-molecular interactions are assessed by comparing the magnitude and sign of these values. The relative coverages of the two components in the monolayer are assessed from the voltammetric reductive desorption.^39^ The theoretical reductive charge is 70 μC cm^−2^, corresponding to the 7.2 × 10^−10^ mol cm^−2^ coverage for a fully formed monolayer (√3 × √3)R30° on gold.^40^ The fractional coverage of two components in the monolayer controlled by the precursors in the solution. A small fraction of double-layer charge in the overall reductive charge has little impact on the analysis of the fractional coverage (*θ*) since the double-layer charges are largely canceled in the coverage calculation.

We systematically determined the coverages of several two-component monolayer assemblies on Au(111) surfaces (*θ*_1_ and *θ*_2_) as a function of concentrations in the solution (*C*_1_ and *C*_2_) by voltammetric measurements, including:

(1) TP/ET monolayer assembly, where TP (thiophenol) features an aromatic moiety while ET (ethanethiol) features a hydrophobic end group with approximately similar molecular length as TP (Fig. S1A†);

(2) MUA/HDT monolayer assembly, where MUA (11-mercaptoundecanoic acid) features an alkyl chain with –CO_2_H end group whereas HDT (hexadecanethiol) features a longer alkyl chain with a hydrophobic –CH_3_ group (Fig. S1B†);

(3) FTP/MHA monolayer assembly, where FTP (4-fluorothiophenol) features an aromatic moiety with a neutral F-group whereas MHA (16-mercaptohexdecanoic acid) features a long-alkyl chain with –CO_2_H groups in the monolayer (Fig. S2A†);

(4) MBA/MHA monolayer assembly, where MBA (mercaptobenzoic acid) maintains the same aromatic structure as FTP but introduces a –CO_2_H group whereas MHA features a long-alkyl chain host-like molecules with –CO_2_H end groups in the monolayer (Fig. S2B†).

Based on simulations using Frumkin isotherms,^36^ the faradaic current contributes largely to the total integrated charge, whereas the capacitive current produces a charge less than 10–20% of the total charge. The latter is supported by a double-layer charge of 15–20 μC cm^−2^ at the desorption potential. Also, in agreement with previous simulations,^41,42^ the width and potential are dependent on the adsorbate–adsorbate interactions, which constitutes the basis for assessing the thermodynamic correlation between surface coverages and the relative amounts of the two precursors in solution. By analysis of the reductive desorption potentials (*E*_p_), we observed an excellent agreement between the DFT calculated binding strength and the reductive desorption potential in terms of their relative differences (see Fig. 1A(b) and S3†).

As shown in Fig. 1B, the general agreement for the fitting of the predictions by eqn (3) to the electrochemical data for the monolayer mixed with TP and ET (Fig. S1A†) establishes the thermodynamic correlation between the surface and solution species. The fitting parameters for TP (1) and ET (2) yield *α*_a_ = 0.05, *α*_b_ = 2.05, and *α*_c_ = −2.0. In comparison, a similar agreement between the theoretical prediction and experimental data is also found for monolayers of longer-chain MUA(1)/HDT(2) system (Fig. S1B†), yielding *α*_a_ = 2.8, *α*_b_ = 2.5, and *α*_c_ = 0.3 (Fig. 1C). In addition, the analysis of the isotherm for mixing short and long RSHs with distinctive structural difference but the same functional groups, *e.g.*, MBA/MHA (Fig. S2B†), yields *α*_a_ = 3.8, *α*_b_ = 2.9, and *α*_c_ = 0.9 (Fig. 1D), displaying a clear shift of the cross point to a higher *C*_1_/*C*_2_ ratio (∼25). The results demonstrate *α*_22_ > *α*_11_ > *α*_12_.

The fact that the competitive adsorption theory fits well with the two-component thermodynamic description rules out the presence of disulfides. Based on *α*_22_ > *α*_11_, the attractive interactions between alkylthiolate-adsorbates are stronger than those between benzylthiolate-adsorbates, and the attractive interactions between long chains are larger than those between short chains, leading to *K*_2_ being greater than *K*_1_. This is consistent with the narrower desorption wave for stronger attractive interactions than those with weak ones for the mixed monolayers in Fig. S1 and S2.† The effect of the intermolecular interactions on the overall binding strength is in fact consistent with the shift in *E*_p_*vs.* mixing ratio.

Considering the summative nature of *α*_a_, *α*_b_, and *α*_c_ in terms of *α*_11_, *α*_22_, and *α*_12_, the finding that *α*_c_ = −2.0 (Fig. 1B) means that TP–TP interaction is smaller than TP–ET interaction, reflecting the dominance of van der Waals interaction in the mixed monolayer, as supported by the small value of *α*_a_. For MUA/HDT and MBA/MHA, *α*_c_ values are not negative but quite small (0.3 and 0.9), suggesting again the important role of van der Waals interactions, a detailed insight into which may be provided by molecular dynamics modeling in the future work.

### Electrochemical perforation

#### (A) Component removal and refill

**Fig. 2:**
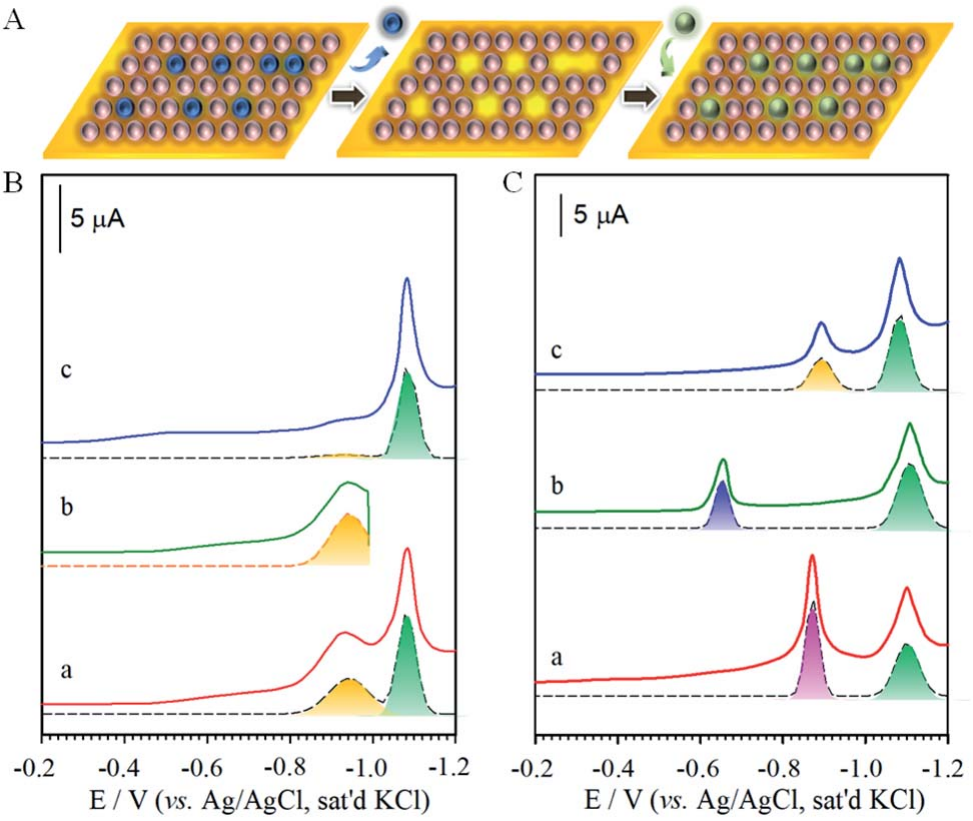
(A) A schematic illustration of electrochemical selective desorption (perforation) from a two-component monolayer on gold. (B and C) Voltammetric curves for MUA/HDT (MUA-to-HDT ratio ∼ 5 with a total thiol concentration of 1.1 mM) in the removal of MUA and refill: (B) MUA/HDT (a), (b) desorption of MUA, and (c) desorption of HDT after b, (C) desorption after refilling B-b with (a) S^2−^, (b) MPA, and (c) MUA (5 mM S^2−^ (Na_2_S), 5 mM MPA (mercaptopropionic acid), or 0.7 mM MUA were used for refilling for ∼10 min. Electrolyte: 0.5 M KOH, geometric surface area of the electrode: 0.6 cm^2^, and scan rate: 50 mV s^−1^).

To achieve well-defined voltammetric deposition waves with a low effective level of a defect-associated current, annealing temperature was systematically varied from room temperature to 400 °C, we found that gold films annealed at ∼300 °C exhibit the optimal performance based on comparisons of leakage current and desorption voltammetric waves. For electrochemically-driven selective desorption for MUA/HDT (Fig. 2A), the desorption of MUA is clearly not detectably destructive to the architecture defined by HDT monolayer (Fig. 2B). Two partially overlapping waves are evident (a) where the more positive wave is for the desorption of MUA and the more negative is for that of HDT. By sweeping from −0.2 to −1.0 V and holding the potential at −1.0 V while agitating the solution, MUA is desorbed (b). This is substantiated by the post-MUA-desorption wave (c), showing that the wave for MUA is virtually undetectable after its selective desorption in (b). The wave in (c) is effectively the same as the more negative one in (a). As shown by the general similarities of the two major waves in the monolayer for the other combinations (TP/ET and MBA/MHA) (Fig. S1 and S2†), the changes in their reductive desorption curves are basically similar to those in Fig. 2B.

The structural integrity of the longer chain component after perforation is supported by the similarities in peak width, peak potential and integrated charges for the wave in Fig. 2B. After desorbing MUA, the integrated charge for the remaining HDT is estimated at ∼23 μC, demonstrating the maintenance of structural integrity of HDT component in the MUA/HDT assembly after perforation, which stems from the strong cohesive intermolecular interactions. We further examined whether the resulting channels from the desorption of MUA in MUA/HDT could be refilled by testing three different species: S^2−^ (a), MPA (b) and MUA (c) (Fig. 2C). In each case, two desorption waves are observed prior to the onset of solvent reduction. The more negative wave corresponds to that for the desorption of HDT, whereas the more positive waves reflect the desorption of the reconstituting adsorbate.

#### (B) Spectroscopic characterizations

**Fig. 3:**
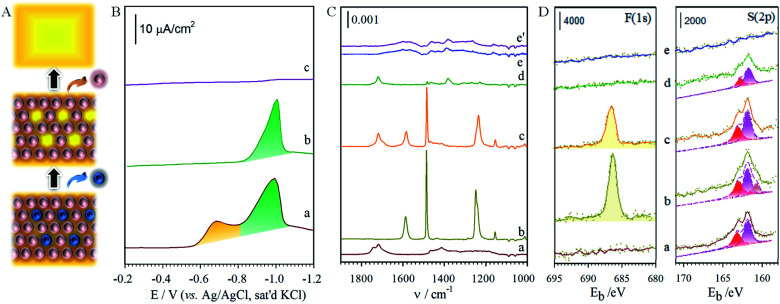
(A) Illustration of selective and sequential removal of FTP and MHA. (B) Voltammetric curves: (a) FTP/MHA (*E*_p (FTP)_ = −0.68 V and *E*_p (MHA)_ = −1.0 V, area ratio of FTP/MHA: 0.51), (b) after holding potential at −0.8 V, and (c) at −1.2 V (electrolyte: 0.5 M KOH, geometric surface area of the electrode: 0.6 cm^2^, and scan rate: 50 mV s^−1^). (C) IRRAS (infrared reflection absorption spectroscopy) for monolayers of MHA (a), FTP (b), and FTP/MHA (c), FTP/MHA after treatment at −0.8 V (d), FTP/MHA after treatment at −1.2 V (e). (e′) is for an ethanol-rinsed, freshly-prepared bare gold that was exposed to the ambient condition. (D) XPS spectra (X-ray Photoelectron Spectroscopy) in the region of F(1s) and S(2p) for monolayers of MHA (a), FTP (b), FTP/MHA in (c), after treatment at −0.8 V (d), and after treatment at −1.2 V (e).

The effectiveness of removing one component without affecting the other component in the monolayer was further demonstrated by spectroscopic characterizations of FTP/MHA. To remove the FTP-component in the mixed monolayer (Fig. 3A), the applied potential was held at the midpoint between the two desorption waves in Fig. 3B(a) (*i.e.*, −0.8 V) for ∼2 min while agitating the electrolytic solution with a stream of argon. The subsequent scan (Fig. 3B(b)) is devoid of FTP-desorption wave, while the wave for MHA-desorption remains largely unchanged. Upon removing both FTP and MHA, the voltammetric curve is devoid of any waves (Fig. 3B(c)).

To pin down the identity of each voltammetric curve, IRRAS (Fig. 3C) and XPS (Fig. 3D) characterizations were performed. The results strongly support the conclusions developed from the electrochemical experiments on sequential desorption. Fig. 3C presents the IRRAS spectra (1900–1000 cm^−1^) obtained for the FTP/MHA samples prepared for the two-component solution and the two steps in the sequential desorption processes by an applied potential at −0.80 and −1.20 V. Spectra for samples formed from the one-component solutions of MHA (a) and FTP (b) are included for comparison. The presence of the FTP-derived component in the mixed monolayer is confirmed in (c) by the bands diagnostic of an aromatic ring (*ν*(C=C) at 1585 and 1487 cm^−1^) and a fluorine functionality (*ν*(C–F) at 1240 cm^−1^); the presence of the MHA-component of the monolayer is supported by the envelope of bands indicative of a carboxylic acid functionality (*ν*(C=O)) between 1740–1690 cm^−1^. By comparison, the virtual absence of the *ν*(C=C) and *ν*(C–F) bands and the persistence of the *ν*(C=O) envelope in spectrum (d) indicate that holding the applied potential at −0.80 V effectively removes all of the FTP-component from the two-component monolayer from the gold surface. The change in the *ν*(C=O) envelope reflects a rearrangement in MHAs is the adlayer that is caused by the removal of FTP from the monolayer and is consistent with the changes in the spectral features for the methylene modes in the C–H stretching region. The *ν*(C=O) peak in (d) is not detected in (e), supporting the removal of MHA at −1.20 V. The residual features in (e) are similar to that obtained for a freshly prepared gold after rinses with ethanol and a brief exposure (a few seconds) to the ambient environment (e′).

The same conclusions are reached by XPS characterization, as shown in F(1s) and S(2p) regions in Fig. 3D for the monolayers prepared from the one-component solutions of MHA (a) and FTP (b) and for the monolayer formed from the two-component solution of MHA and FTP before (c) and after electrochemical manipulation (d: −0.80 V, and e: −1.20 V). In general, the spectral data for the monolayers prepared from the one- and two-component solutions are consistent with the expected compositions. That is, only spectra (b) and (c) have detectable bands in the F(1s) region, with the lower intensity of the band in spectrum (c) relative to (b) reflecting the lower coverage of the FTP-component in the two-component monolayer. The spectra in the S(2p) region are also in general agreement with the expected sample compositions, and are consistent with earlier assignments to gold-bound thiolates.^7,8^ There is, however, an additional piece of structural detail evident in the spectrum for the FTP-derived monolayer in Fig. 3D. The appearance of the lower energy shoulder in (b), as opposed to the usual doublet-shaped peak in (a), reveals the presence of a small relative amount of adsorbed atomic and oligomeric sulfur, as described in the discussion of the voltammetric data of Fig. S2A.† The deconvoluted data included with the data further clarify this interpretation. That is, two S(2p) couplets are evident in (b) and only one couplet in (a). The S(2p) couplet at high energy (S(2p_3/2_) ∼ 162.0 eV and S(2p_1/2_) ∼ 163.2 eV) are assigned to sulfur bound to gold as a thiolate and the S(2p) couplet at (S(2p_3/2_) ∼ 161.1 eV and S(2p_1/2_) ∼ 162.2 eV) to sulfur bound to gold in its atomic and oligomeric forms. After treatment at an applied potential of −0.8 V, the absence of a detectable (∼0.1 monolayer detection limit) F(1s) band in Fig. 3D(d) is diagnostic of the removal of the FTP component from the monolayer. The decrease of the intensities of the S(2p) couplet in spectrum (d) relative to (c) also supports the decrease in the total sulfur coverage at the gold surface. Furthermore, the absence of a detectable sulfur S(2p) peak for the sample after the −1.2 V-treatment (e) reflects the exhaustive removal of the remaining portion of the original mixed monolayer, and is in strong agreement with the electrochemical and IRRAS data in Fig. S2A† and 3C. This analysis proceeds by comparing the integrated band intensities for the deconvoluted S(2p) couplets of the thiolate species before and after the removal of the FTP-derived component, which were normalized using the intensities of the bands for the corresponding single-component spectra to estimate for the effects of attenuation. This analysis yields a mixing ratio of FTP to MHA ∼ 0.43, in close agreement with the value determined from the electrochemical data.

### Nanoelectrode arrays

#### (A) Voltammetric characteristics

**Fig. 4:**
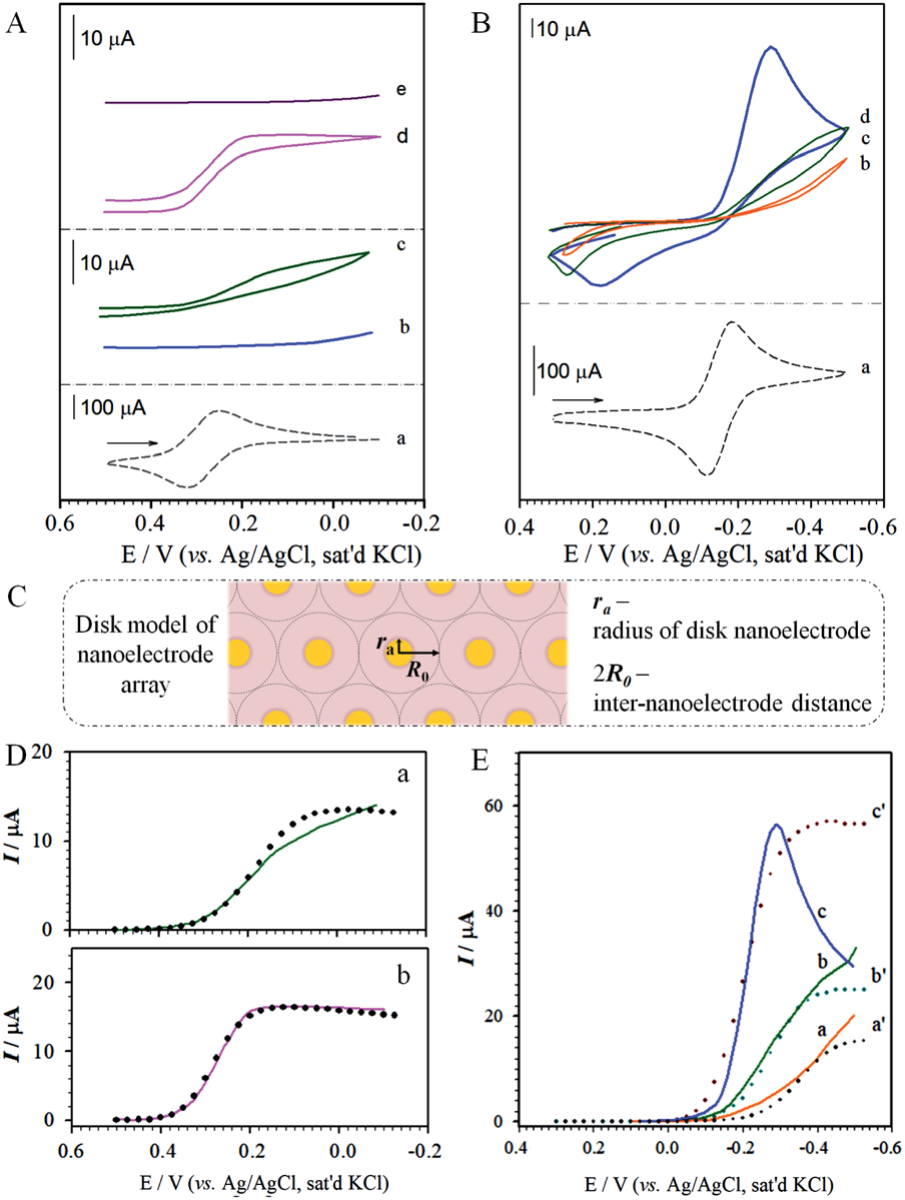
(A, B) Cyclic voltammetric curves: (A) Fe(CN)_6_^3−^ on (a) gold; (b, c) MUA/HDT (before (b) and after (c) removal of MUA); and (d, e) perforated MBA/MHA at pH ∼ 3.5 (d) and pH ∼ 10.5 (e) (electrolyte: 1 M KCl solution containing 1.3 mM Fe(CN)_6_^3−^, pH ∼ 7, scan rate: 50 mV s^−1^). (B) Ru(NH_3_)_6_^3+^ on gold (a, scan rate: 50 mV s^−1^), perforated MBA/MHA in 1.0 M KCl/1.3 mM Ru(NH_3_)_6_^3+^ at pH ∼ 3.5 (b), pH ∼ 6.0 (c), and pH ∼ 10.0 (d) (scan rate: 20 mV s^−1^). (C) theoretical disk-model of nanoelectrode array (drawn not to scale). (D, E) Comparison of the experimental (solid lines) with simulation results (dotted lines) for A-c using *r*_a_ = 2.0 nm, *R*_0_ = 482 nm, *D* = 3.20 × 10^−6^ cm^2^ s^−1^, and *k* = 2 (D, a); for A-d using *r*_a_ = 20 nm, *R*_0_ = 1950 nm, *D* = 3.20 × 10^−6^ cm^2^ s^−1^, and *k* = 2 (D, b); and for B using *r*_a_ = 0.7 nm, *R*_0_ = 545 nm, *D* = 8.43 × 10^−6^ cm^2^ s^−1^, and *k* = 2 at pH ∼ 3.5 (E, a); *r*_a_ = 7.5 nm, *R*_0_ = 2160 nm, *D* = 2.0 × 10^−5^ cm^2^ s^−1^, and *k* = 2 at pH ∼ 6.0 (E, b); and *r*_a_ = 600 nm, *R*_0_ = 5.70 × 10^4^ nm, *D* = 3.0 × 10^−4^ cm^2^ s^−1^, and *k* = 2 at pH ∼ 10.0 (E, c).

It is well established that non-linear diffusion can be induced by a partial blockage of an electrode surface towards electron transfer,^37^ where microelectrode phenomena^38^ may appear when the average size of the active sites is very small and the average distance between the unblocked areas is relatively large. These microelectrodes are of molecular or nano-dimensions in size. The access of redox probes to the underlying electrode may thus be externally gated by pH-tuning of the terminal group between protonated carboxylic acid form and negatively-charged carboxylate form, leading to a change in electrostatics around the rim of the channel. This is demonstrated with two ionic redox probes, Ru(NH_3_)_6_^3+/2+^ and Fe(CN)_6_^3−/4−^. Fig. 4A(b) and (c) shows the results for Fe(CN)_6_^3−^ on the perforated MUA/HDT monolayer before (b) and after (c) the removal of the MUA component. This characteristic is independent of pH. In comparison with Fe(CN)_6_^3−^ on a bare Au(111) (a), the results on the perforated MBA/MHA monolayer (Fig. 4A(d) and (e)) show a clear dependence on pH, exhibiting sigmoidal current at low pH (d) and diminished current at pH above 10 (e). This result is consistent with protonation/deprotonation of –CO_2_H groups of the MHA skeleton at the rim of the microchannels. While MBA/MHA assembly acts as an effective barrier between the redox probe and the underlying substrate, the perforated assembly after removal of MBA exhibits a pH-tunable sigmoidal-shaped voltammetric curve (c) for Fe(CN)_6_^3−^, which is characteristic of radial diffusion of a microelectrode array or ensemble.^43^ The half-wave potential for curve (b) is similar to that estimated from the formal reduction potential for Fe(CN)_6_^3−/4−^ (a).

The dependence on pH for the positively-charged redox probe, Ru(NH_3_)_6_^3+^, is shown in Fig. 4B. The voltammetric curve of the redox couple at a bare gold electrode is included for comparison (a). The key observation is that the redox current increases upon increasing pH (b–d), a finding not observed prior to perforation. This result is consistent with the expectation based on the pH-induced electrostatic gating mechanism. We also note that the voltammetric curves show peak-shaped and widely-separated redox waves that are not characteristic of a radial diffusion-controlled process. We attribute this result to a significant reduction of the apparent heterogeneous electron transfer rate constant (k^app^_s_) for the redox probe at the monolayer-assembled electrode.^37^ Indeed, an estimate of k^app^_s_ based on the peak separation^44^ at pH 10 (Δ*E*_p_ ∼ 500 mV) yields a value of 5.9 × 10^−4^ cm s^−1^, which is at least three orders of magnitude lower than that for the solution-phase rate constant. Further investigations are needed to gain an improved understanding of this observation.

#### (B) Theoretical modeling

To provide a more quantitative description of the nanoelectrode array formed by electrochemical perforation, the number of microchannels in the array can be determined from the fractional coverage of the desorbed component (*θ*_μ_) or the non-desorbed component (*θ*_block_) (*θ*_μ_ + *θ*_block_ = 1) by integrating the charge under each of the desorption waves by *θ*_μ_ = *Q*_μ_/(*Q*_μ_ + *Q*_block_), where *Q*_μ_ is the charge for the desorbed component upon perforation, and *Q*_block_ corresponds to the charge for the non-desorbed component. Experimentally, however, an extremely small fraction (*i.e.*, *θ*_μ_ = ∼0.02) of the shorter length component is mixed into the monolayer in order to generate highly dispersed nanoelectrodes which is undetectable in the desorption voltammetry. For this reason, we estimate the surface density of the microelectrode-like channels based on models of microelectrode arrays, either disk-shaped microelectrodes with an average radius of *r*_a_, or strip-shaped microelectrodes with an average width of *r*_a_ (Scheme S1†). The charge transfer is considered to occur at a partially blocked electrode surface,^36^ and has been applied to the simulations of defects in monolayer systems. In an idealized model, 2*r*_a_ and 2*R*_0_ represent the average diameter of the perforated electrodes and the average distance between them (Fig. 4C). If the fractional coverage of the blocking architecture (*θ*_block_) is close to unity, *i.e.*, *θ* → 1, the voltammetric current plateau (*I*_lim_) of the array can be expressed as:

4 *I*_lim_ = *nFADC*^0^/[2*R*_0_*f*(1 − *θ*)]

where *n* is the electron transfer number, *F* is the Faraday constant, *A* is the electrode surface area, *D* is the diffusion coefficient, and *C*^0^ is the concentration of the redox probe; the coverage function, defined as *f*(1 − *θ*), equals 0.3(1 − *θ*)^−1/2^. In the model (Scheme S1†), the electrode radius (*r*_a_) − interelectrode distance (2*R*_0_) are related to the surface coverage of the electrodes (1 − *θ*) by *r*_a_ = *R*_0_ (1 − *θ*)^1/2^ (disk-type), or *r*_a_ = *R*_0_ (1 − *θ*) (strip-type). The dimensionless equation of current (*i*)∼potential (*E*) (see ESI†) depends on the function of *f*(1 − *θ*), numerical solution of which is used for the simulations of the experimental data in terms of the nanoelectrode parameters.

Fig. 4D(a) shows the theoretical modeling results with the disk-model for the reduction of Fe(CN)_6_^3−^ on the perforated MUA/HDT. The data showed an approximate fit to the experimental data at potentials greater than 0.1 V, revealing *r*_a_ = 2.0 nm, *R*_0_ = 482 nm. The poor fitting at potentials below 0.1 V is believed due to the presence of leakage current at the perforated MUA/HDT. The values of *D* and *k* are quite consistent with the literature report.^45,46^ The nanoelectrode array features disks with about 2.0 nm in radius and about 960 nm (2*R*_0_) in nearest-neighbour distance. The observation of the radial diffusion current is also expected considering the size of Fe(CN)_6_^3−^ which is 0.4–0.5 nm. For the theoretical modeling result for the reduction of Fe(CN)_6_^3−^ on the perforated MBA/MHA (Fig. 4D(b)), the disk-model showed an excellent fit to the experimental data in the entire potential range. The nanoelectrode array features disks with about 20 nm in radius and about 3900 nm in the nearest-neighbor distance. Apparently, the nanoelectrode is 10× larger and the inter-disk spacing is 4 times greater than those for the perforated MUA/HDT, showing 12× higher active area. Note that the strip-model did not show any good fit to the experimental data for both perforated MUA/HDT and MBA/MHA (see Fig. S4†).

Fig. 4E shows the theoretical modelling results with the disk-model for the reduction of Ru(NH_3_)_6_^3+^ at three different pH values on the perforated MBA/MHA. In these cases, the disk-model simulation result showed fitting to the experimental data at potentials before reaching current-peak/plateau potentials, yielding *r*_a_ = 0.7 nm and *R*_0_ = 545 nm for pH ∼ 3.5 (a); *r*_a_ = 7.5 nm and *R*_0_ = 2160 nm for pH ∼ 6.0 (b); and *r*_a_ = 600 nm and *R*_0_ = 5.70 × 10^4^ nm for pH ∼ 10.0 (c). In comparison with the *D* value for Fe(CN)_6_^3−^ on the perforated MBA/MHA (Fig. 4D(b)), the value of *D* for the reduction of Ru(NH_3_)_6_^3+^ is increased, which is somewhat surprising. Since the diffusion coefficient *D* is referred to near the electrode surface, it is not totally unreasonable considering similar values reported in the literature,^47^ and the strong electrostatic attraction by the positive redox probe's charge and the negative charge (–CO_2_^−^ groups of MHA) at the entrance of the nanoelectrode channel. This interpretation however needs further confirmation.

At pH ∼ 6.0, the nanoelectrode array features disks with about 8 nm in radius and about 4320 nm in nearest-neighbour distance. The radius is increased by a factor of 80 at pH ∼ 10.0 but reduced by a factor of ∼10 at pH ∼ 3.5. The disk radius at pH ∼ 10.0 is much larger than the radius of Ru(NH_3_)_6_^3+^ (∼0.4 nm), resulting in a peak-shaped voltammetric wave. Interestingly, the *D* value is shown to increase with pH, coinciding with the increase of the electrostatic attraction at higher pH. Again, the experimental data cannot be fitted using the strip-model (see Fig. S5†).

While the nanoelectrode pore size, shape and interpore distance are derived from the theoretical simulation, there have been extensive AFM (atomic force microscopy) studies of SAMs or mixed SAMs,^6,7,14,48^ though with limited resolution under ambient condition, for measuring the pore sizes. One relevant example involves AFM study of mercaptopropionic acid (MPA)–MUA monolayer assembled from 1 : 4 ratio of MPA : MUA.^49^ The monolayer after reductive desorption of MPA showed nanopores with diameters of 12–19 nm, supporting the formation of nanopores in the monolayer which is similar to our MBA/MHA monolayer. Moreover, in a recent field ion microscopy (FIM) study of a mixed monolayer of octanethiol (ODT) and perfluorodecanethiol (PFDT),^50^ PFDTs are shown to arrange in a patchy type of domains surrounded by ODTs due to the cross intermolecular interaction with the homogeneous inter-chain interaction energy being larger than the cross-interaction energy, supporting our assessment of the homo and hetero-intermolecular interactions.

Consider further the array performance by analyzing the limiting current (*i*_lim_) for the radial diffusion at a microelectrode,^43^ which is given by:

5 *I*_lim_ = *N*_μ_*i*_lim_ = *N*_μ_(4*nFDC*^0^*r*_a_)

where *N*_μ_ is the number density of nanoelectrodes. The total limiting current (*I*_lim_) density is the sum of the limiting current from all individual nanoelectrodes.^43^ Based on eqn (5), the theoretical single nano-electrode limiting current is calculated (*i*_theory_). As shown in Table S2,† the result is quite comparable with the simulation results (*i*_simulated_) after taking the number of nanoelectrode into consideration (*N*_μ_). The *i*_theory_ and *i*_simulated_ values are quite comparable (Table S2 and Fig. S6†), showing almost identical values in the case of Fe(CN)_6_^3−^ on perforated MHA/HDT and MBA/MHA. The *i*_simulated_ value is slightly smaller than *i*_theory_ in the case of Ru(NH_3_)_6_^3+^ on perforated MBA/MHA.

**Scheme 1:**
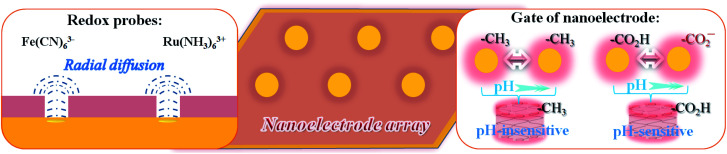
Illustrations of radial diffusion as the nanoelectrode arrays, and pH-sensitive and insensitive interfacial characteristics.

The above results have demonstrated the general micro/nano electrode characteristics of the nanoelectrode arrays,^43^ which not only show the radial diffusions at the nanoelectrode arrays, but also the characteristics of pH-insensitive and pH-sensitive interfaces for the arrays (Scheme 1). Considering the Debye length at the electrolyte concentration,^44^ the fixed negative charges of the carboxylate groups are effectively screened by cations at a distance of a few angstroms, which is reasonably consistent with the average diameters of the redox probes. This nanoelectrode array or ensemble indeed features molecularly- and chemically-tunable characteristics.

## Conclusions

Taken together, the intermolecular interactions are shown to play a critical role in creating the nanoscale channels with nanoelectrode functionality. The strategy of electrochemical perforation by rational combinations of homo-/hetero-intermolecular interactions not only opens an intriguing avenue for the creation of nanochannel-like nanoelectrode at a molecular level, but also offers the capacity for manipulation of the nanoelectrode properties by refilling the perforation sites with a pre-selected molecular functionality or dimension. The array or ensemble of such nanoelectrodes is potentially capable of functioning as an artificial membrane for molecularly-controllable and tunable ion-gating, which could lead to a paradigm shift in the design of biosensors.

## Experimental section

### Chemicals

Thiophenol (TP), 4-fluorothiophenol (FTP), mercaptobenzoic acid (MBA), ethanethiol (ET), hexadecanethiol (HDT), mercaptopropionic acid (MPA), 11-mercaptoundecanoic acid (MUA), 16-mercaptohexdecanoic acid (MHA), and 1,4-mercaptobenzoic acid (MBA) were purchased from Aldrich. Potassium ferricyanide (99+%), hexaamineruthenium(iii) chloride (98%), potassium chloride (99+%), hydrochloric acid (ACS reagent), sodium sulphide (Na_2_S, ACS reagent), and potassium hydroxide (semiconductor grade, 99.99%) were used as received from Aldrich. Water was purified with a Millipore Milli-Q system.

### Sample preparation

The substrates were prepared by the vapor deposition of 300 nm of gold (99.9% purity) onto freshly cleaved mica sheets (B & M Trading Inc.) or pre-cleaned glass slides using an Edwards 306A cryopumped evaporator. The glass slides were primed with 15 nm of chromium prior to gold deposition. In both cases, gold was deposited at a rate of ∼0.3 nm s^−1^. The pressure in the evaporator was less than 1 × 10^−6^ Pa before deposition. The mica-supported gold films were subsequently annealed at 300 °C for ∼4 h, a process that yields a surface composed of large (100–200 nm^2^) atomically smooth Au(111) crystallites. The roughness factor, as determined by a comparison of the surface area from the oxidative stripping of iodide to the exposed geometric area of the electrode, is 1.1 ± 0.1. Monolayers were formed by immersing the gold substrates into ethanolic solutions with a controlled thiol concentration. Typically, the monolayers were obtained using thiol concentrations of >1 mM, assembly time of >12 hours and room temperature (25 °C), which are important for achieving not only thermodynamic equilibrium but also better molecular packing in the SAMs. The use of 5 mM total concentration is for the purpose of well-defined thermodynamic analysis by varying the mixing ratio of the two components while fixing the total concentration. All samples were thoroughly rinsed with ethanol and dried under argon before characterization. We also note that the reduction peak can be displayed on polycrystalline gold surface without annealing to form Au(111), but the multiple peaks due to the presence of multiple facets (*e.g.*, 111, 110, 100)^41^ complicate the controllability. Polycrystalline gold could be used if the first component's desorption is separated from the second one.

### Instrumentation

Electrochemical measurements were performed using a CV-27 potentiostat, including electrochemical reductive desorption^51^ and cyclic voltammetry. A conventional three-electrode cell was employed, with a platinum coil as auxiliary electrode and an Ag/AgCl (sat'd KCl) electrode as reference electrode; all potentials are given with respect to this reference electrode. The geometric area of the working electrode, 0.60 cm^2^, was defined by an O-ring which defines the diameter on the working electrode (0.88 cm). The supporting electrolyte was an aqueous solution of KOH (0.5–1.0 M) that was deaerated with ultrahigh purity argon before electrochemical measurements. The pH of the solution was controlled by adding aliquots (a few μL) of 1.0 M HCl or 1.0 M KOH into the solution while maintaining the high ionic concentration (1.0 M KCl). For the coverage analysis, the voltammetric peaks and integrated charges were determined by deconvolution with mixed Lorentzian–Gaussian curves. The measurement of the reductive desorption potential is reproducible within ±5%, and the integrated desorption charge is reproducible within ±10%.^42^

Infrared reflection absorption spectroscopy (IRRAS) was acquired with a Nicolet 760 ESP FTIR that was purged with boil-off from liquid nitrogen and equipped with a liquid nitrogen-cooled HgCdTe detector. The spectra were obtained in an external reflection mode using p-polarized light incident at 80° with respect to the surface normal, and with 500 scans at 2 cm^−1^ resolution. An octadecanethiolate-d_37_ monolayer was used as the reference.

A Physical Electronics Industries 5500 surface analysis system was used for the X-ray Photoelectron Spectroscopy (XPS) characterizations. This system is equipped with a hemispherical analyzer, toroidal monochromator, and multichannel detector. The sampling area was ∼2 mm^2^. A pass energy of 29.35 eV was used with a resolution of ∼0.3 eV. Monochromatic Al K_α_-radiation (1486.6 eV) at 300 W was used for excitation. Photoelectrons were collected at 45° from the surface normal with acquisition times less than 10 min. The Au(4f7/2) emission band served as an internal reference for binding energies. The base pressure of the XPS chamber was less than 9 × 10^−10^ torr during all analyses. The XPS spectral couplet in the S(2p) region was fitted using a doublet with a Gaussian profile. This doublet is the result of spin–orbit coupling which splits the S(2p) band into 2p_3/2_- and 2p_1/2_-components separated by 1.2 eV with half-height widths of 0.9 ± 0.05 eV, differing in intensity by a factor of two (2p_3/2_ > 2p_1/2_).^52^

## Author contributions

HWC carried out the data analysis and theoretical simulation, and performed part of the data acquisition. SW carried out part of the simulation and data analysis. MDP and CJZ conceived and supervised the research. CJZ designed the experimental measurement and theoretical simulation, and directed the manuscript writing and revision. All co-authors assisted in writing and revising the manuscript. All co-authors read and approved the final manuscript.

## Conflicts of interest

There are no conflicts of interests to declare.

## Supplementary Material

SC-012-D0SC06955H-s001
