# Supplementary material for: Molecularly-tunable nanoelectrode arrays created by harnessing intermolecular interactions
Source: Chem Sci. 2021 Mar 10;12(17):6081–90. doi: 10.1039/d0sc06955h (PMC8098684; doi:10.1039/d0sc06955h)
Supplement: SC-012-D0SC06955H-s001 [file SC-012-D0SC06955H-s001.pdf]

This journal is © The Royal Society of Chemistry 2021

Electronic Supplemental Information

Molecularly-Tunable Nanoelectrode Arrays Created by Harnessing Intermolecular Interactions

Han-Wen Cheng[a,b]*, Shan Wang[b], Marc D. Porter[c]*, and Chuan-Jian Zhong[b]*

a) School of Chemical and Environmental Engineering, Shanghai Institute of Technology, Shanghai 201418, China
b) Department of Chemistry, State University of New York at Binghamton, Binghamton, New York 13902, USA
c) Department of Chemistry and Chemical Engineering, University of Utah, Salt Lake City, Utah 84112, USA

Additional experimental data and data analysis:

Table S1 Summary of the binding strength estimated based on density functional theory (DFT)-calculated E_b*[1,2] for the selected thiolate molecules ($CH_3(CH_2)_{n-1}SH$) on gold surfaces.

Thiolate molecules	E_b* (eV)	
FTP	-3.43	Linear extrapolation based on "electronegativity *vs*. E*" ($E_b = -0.6449\times n -0.8533$)
MHA	-4.39	Linear regression based on Ref. [1] ($E_b = -0.1043\times n -2.6286$)
MBA	-2.56	Ref. [2]
TP	-2.20	Ref. [2]
ET	-2.61	Ref. [2]
MUA	-3.48	Linear regression based on Ref. [1] ($E_b = -0.1043\times n -2.6286$)
HDT	-4.0392	Linear regression based on Ref. [1] ($E_b = -0.1063\times n -2.3384$) [2]

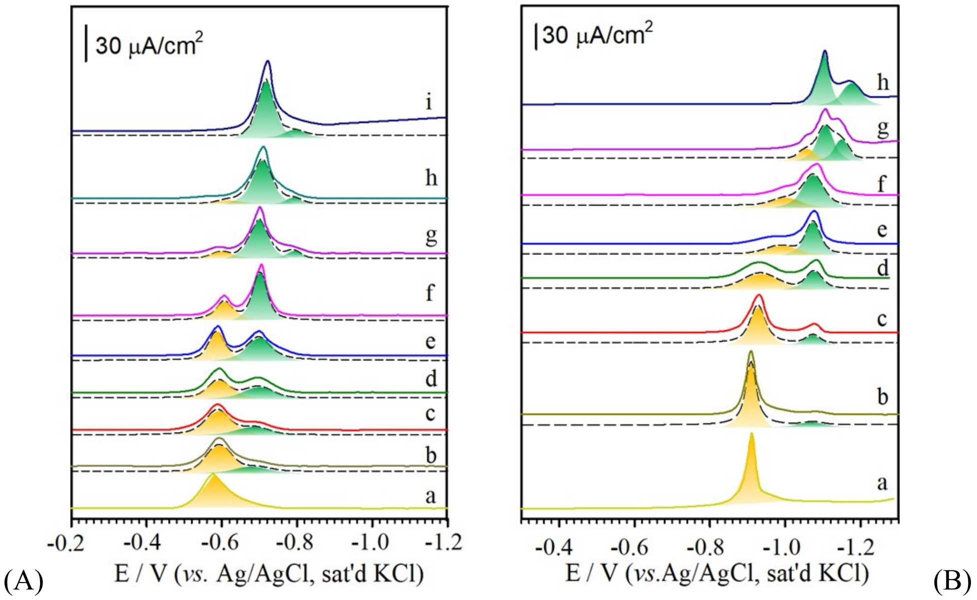

Fig. S1 Voltammetric curves (dashed and colored curves represent deconvoluted waves). (A) TP/ET: (a) TP (E_p= -0.58 V). (b), (c), (d), (e), (f), (g), and (h) TP/ET (mixing ratios: 30, 15, 10, 5, 2, 0.5, and 0.1 with a total thiol concentration of 5.0 mM. E_p: -0.59 and -0.69 V (b); -0.59 and -0.69 V (c); -0.59 and -0.70 V (d); -0.60 and -0.70 V (e); -0.61 and -0.70 V (f); -0.61, -0.70 and -0.78 V (g) and -0.61, -0.71 and -0.78 V (h)). (i) ET (E_p= -0.72 and -0.78 V). The more negative potential wave corresponds to the monolayer of ET (-0.72 V) which has a narrower width (45 mV) than those for the TP-derived monolayer (-0.58 V, 85 mV) reflects a difference in adsorbate-adsorbate interaction. (B) MUA/HDT: (a) MUA (E_p = -0.89 V). (b), (c), (d), (e), (f), and (g) MUA/HDT (mixing ratios: 18.0, 9.0, 6.0, 3.5, 2.0, and 1.0 with a total thiol concentration of 2.5 mM. E_p: -0.91V and -1.08 V (b), -0.94 and -1.08 V (c), -0.94 and -1.09 V (d), -0.96 and -1.08 V (e), -0.99, and -1.08 (f), -1.05 V, -1.10 and -1.14 V (g)). (h) HDT (E_p: -1.11 and -1.17 V). (Electrolyte: 0.5 M KOH, Geometric surface area of electrode: 0.6

cm^2, scan rate: 50 mV/sec). In comparison with the single wave characteristic at the less negative potential (E_p = -0.89 V) for MUA monolayer, HDT monolayer features a double-wave character at the more negative potential (h), -1.11 V and -1.17 V, which is consistent with earlier finding[3] on the voltammetric desorption potentials as a function of the number of carbons (n) in the alkyl chain.

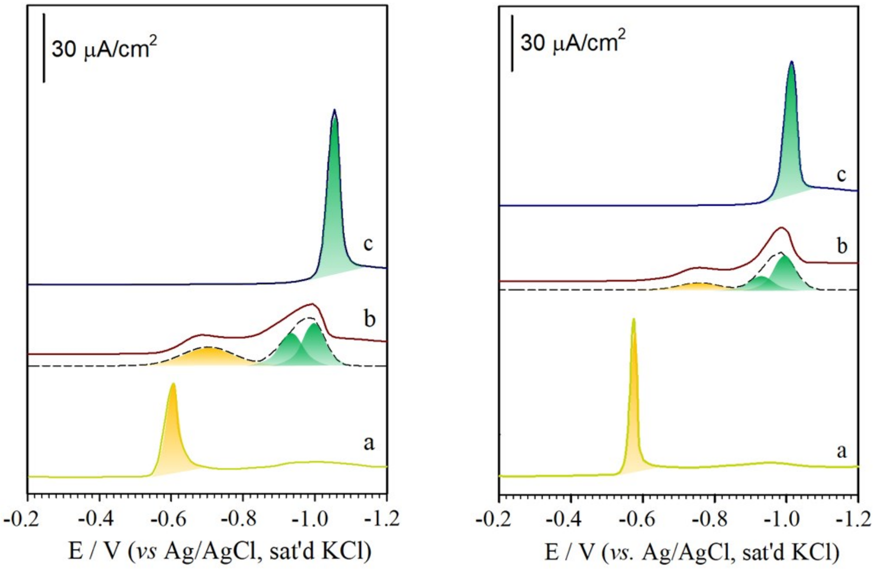

(A) (B)

Fig. S2 Voltammetric curves: (A) (a) FTP (E_p (FTP) = -0.60 V), (b) FTP/MHA (FTP/MHA mixing ratio: 25 with a total thiol concentration of 3.5 mM (E_p (FTP)= -0.68 V and E_p (MHA)= -1.0 V)), and (c) MHA (E_p (MHA)= -1.06 V). (B) (a) MBA (E_p (MBA)= -0.57 V), (b) MBA/MHA (MBA/MHA mixing ratio: 20 and a total thiol concentration of 3.4 mM (E_p (MBA)= -0.78 V and E_p (MHA)= -0.99 V)), and (c) MHA (E_p (MHA)= -1.06 V). (Electrolyte: 0.5 M KOH, Geometric surface area of the electrode: 0.6 cm^2, and Scan rate: 50 mV/sec).

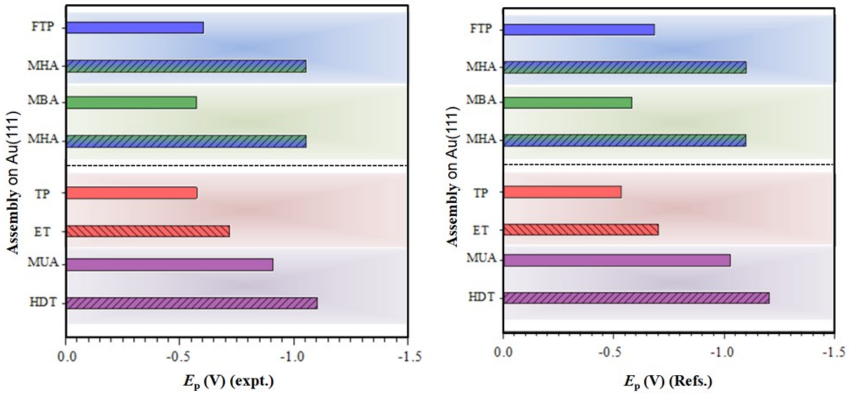

(A) (B)

Fig. S3 (A) Plot of the reductive desorption potentials (E_p) obtained from the experiments for the different pairs of molecules studied in this work. ($E_{p(Ag/AgCl)}$). (B) Reductive desorption potentials (E_p) estimated based on literature reports for different thiolate molecules on gold surfaces: FTP's $E_{p(SCE)}$ = -0.73 V (based on fitting[2]); MHA's $E_{p(SCE)}$ = -1.15 V (based on fitting[2]); MBA's $E_{p(SCE)}$ = -0.63 V[4]; TP's $E_{p(SCE)}$ = -0.58V[5]; ET's $E_{p(SCE)}$ = -0.75 V[6]; MUA's $E_{p(SCE)}$ = -1.08 V (based on fitting[2]); and HDT's $E_{p(SCE)}$ = -1.25 eV (based on fitting[2]). Note that the potential is give in $E_{p(Ag/AgCl)}$, which is - 0.05 V with respect to $E_{p(SCE).}$

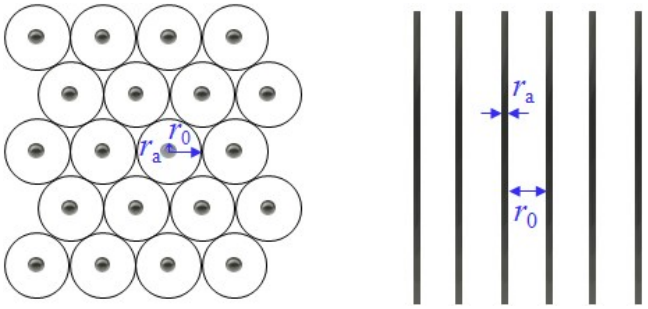

Scheme S1. Simulation models of the nanoelectrode arrays: disk-shaped (A) and strip-shaped (B) models. Simulation of nanoelectrode array is based on the dimensionless equation of current (i) ~ potential (E) derived by Amatore *et al.*[7]**:**

For disk-type:

$$r_a = r_0(1-\theta)^{1/2} \quad (1)$$

For stripe-type:

$$r_a = r_0(1-\theta) \quad (2)$$

Dimensionaless equation of current ~ potential:

$$\Psi = \Lambda(1-\theta)\exp(\alpha\xi)\left\{\left[1 - I\Psi - 2r\Psi f(1-\theta)\right] - \left[I\Psi + 2r\Psi f(1-\theta)\right]\exp(-\xi)\right\} \quad (3)$$

where

$$r = r_a\left(\frac{DRT}{F\upsilon}\right)^{-1/2} \quad (4)$$

$$\Lambda = k_0\left(\frac{RT}{DF\upsilon}\right)^{1/2} \quad (5)$$

$$\xi = -\frac{F}{RT}(E - E^0) \quad (6)$$

$$\tau = \frac{F}{RT}\upsilon t \quad (7)$$

$$\Psi = \frac{i}{FAC^0\left(\frac{DF\upsilon}{RT}\right)^{1/2}} \quad (8)$$

$$I\Psi = \frac{1}{\sqrt{\pi}}\int_0^{\tau}\frac{\Psi}{(\tau-\eta)^{1/2}}d\eta \quad (9)$$

The $f(1\text{-}\theta)$ function is

$$f(1-\theta) = 0.3(1-\theta)^{-1/2} \text{ for disk-type} \quad (10)$$

and

$$f(1-\theta) = 1 - \frac{1}{\sqrt{\pi}}\ln\left[\frac{\pi}{2}\sin(1-\theta)\right] \text{ for stripe-type} \quad (11)$$

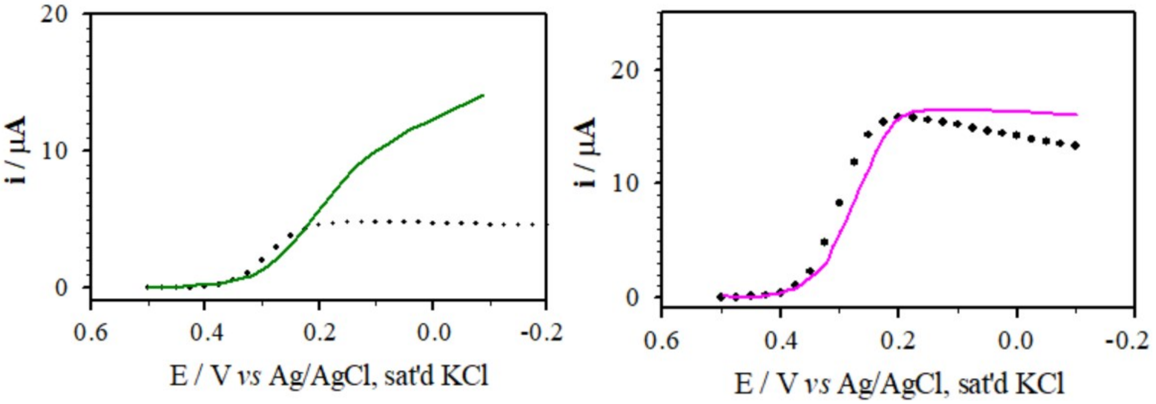

(A) (B)

Fig. S4 Theoretical modeling results for the reduction of $Fe(CN)_6^{3-}$ on the perforated MUA/HDT (A) and MBA/MHA (B). (A) The comparison of experimental data (lines) from Fig. 4A (c) with the simulation (points) in strip-model using r_a= 1.23 nm, R_0= 2.05×10^4 nm ($1\text{-}\theta$ =6.0×10^{-5}), D= 1.12×10^{-6} cm^2/s, and k =2. (B) The comparison of experimental data (lines) from Fig. 4A (d) with the simulation (points) in strip-model using r_a= 2.74 nm, R_0= 6.09×10^3 nm ($1\text{-}\theta$ =5.0×10^{-4}), D= 1.12×10^{-6} cm^2/s, and k =2.

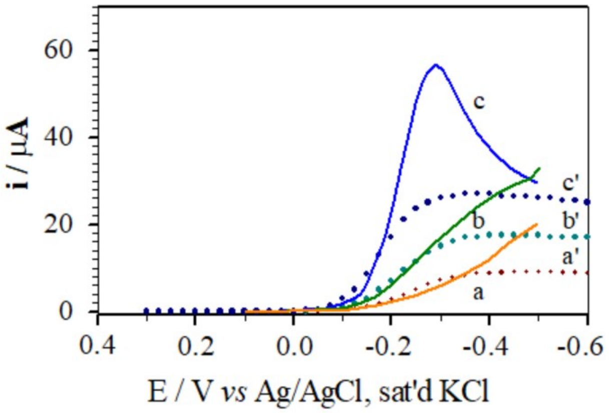

Fig. S5 Theoretical modeling results for reduction of $Ru(NH_3)_6^{3+}$ at three different pH values on the perforated MBA/MHA system. The comparison of experimental data (lines: a, b, and c) from (Fig. 4B (b-d)) with the simulation (points: a', b', and c') in strip-model using r_a= 0.5 nm, R_0= 5.57×10^4 nm ($1\text{-}\theta$ =8.0×10^{-1}), D= 7.5×10^{-6} cm^2/s, and k= 2 for pH~3.4 (a); r_a= 0.9 nm, R_0= 3.75×10^4 nm ($1\text{-}\theta$ =2.5×10^{-5}), D= 1.1×10^{-5} cm^2/s, and k= 2 for pH~6.0 (b); and r_a= 0.4 nm, R_0= 3.75×10^4 nm ($1\text{-}\theta$ =1.03×10^{-4}), D= 1.7×10^{-5} cm^2/s, and k= 2 for pH~10.3 (c).

Table S2. Summary of parameters for nanoelectrode array simulation (disk-model Fig. 4D-E).

	Perforated Film	r_a (nm)	R_0 (nm)	1-θ	i_{theory} (μA)	$i_{simulated}$ (μA)	N_μ
(i) $Fe(CN)_6^{3-}$	MUA/HDT	2	482	8.62×10^{-6}	3.21×10^{-7}	3.08×10^{-7}	4.39×10^7
(ii) $Fe(CN)_6^{3-}$	MBA/MHA	20	1950	1.05×10^{-4}	3.21×10^{-6}	3.07×10^{-6}	5.35×10^6
(iii) $Ru(NH_3)_6^{3+}$	MBA/MHA	7.5	2160	1.21×10^{-5}	7.53×10^{-6}	5.71×10^{-6}	4.38×10^6

Note: Data for i, ii and iii are the data from Fig. 4D (a), 4D (b) and 4E (b). $i_{theory} = 4nFDC^0 r_a$; $i_{simulated} = I_{lim} / \#N$ where $N_\mu = A_{total}/A_{singl} = A_T/\pi R_0^2 = A_T(1\text{-}\theta)/\pi r_a^2$, A_T = 0.64 cm^2. (see Fig. S6 for single nanoelectrode limiting current.)

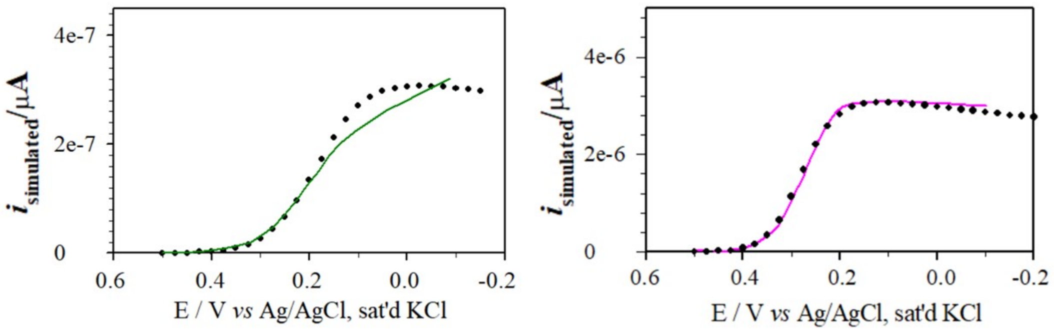

(A) (B)

Fig. S6 Comparison between the experimental (line) and simulated (point, disk-model) steady state limiting current based on eqn. 5. (A) Perforated MUA/HDT, (B) perforated MBA/MHA monolayer (Electrolyte: 1 M KCL with 1.3 mM $Fe(CN)_6^{3-}$).

References:

1. D. Nassoko, M. Seydou, C. Goldmann, C. Chanéac, C. Sanchez, D. Portehault, F. Tielens. Rationalizing the formation of binary mixed thiol self-assembled monolayers, *Mater. Tod. Chem.*, 2017, **5**, 34-42.
2. R. C. Salvarezza, P. Carro. The electrochemical stability of thiols on gold surfaces. *J. Electroanal. Chem.*, 2018, **819**, 234–239.
3. C. J. Zhong, M. D. Porter, The fine structures of voltammetric curves for the electrochemical desorption of organosulfur-based monolayers at gold, *J. Electroanal. Chem.*, 1997, **425**, 147.
4. M. C. R. González, A. G. l. Orive, P. Carro, R. C. Salvarezza, C. Ahn, Structure and electronic and charge-transfer properties of mercaptobenzoic acid and mercaptobenzoic acid–undecanethiol mixed monolayers on Au (111), *J. Phys. Chem.* 2014, **118**, 30013–30022.
5. H. Kang, J. Noh, Influence of thiol molecular backbone structure on the formation and reductive desorption of self-assembled aromatic and alicyclic thiol monolayers on Au(111) surface, *Bull. Kor. Chem. Soc.* 2013**, 34**, 1383–1387.
6. S. Imabayashi, M. Iida, D. Hobara, Z. Q. Feng, K. Niki, T. Kakiuchi, Reductive desorption of carboxylic-acid-terminated alkanethiol monolayers from Au(111) surfaces, *J. Electroanal. Chem*. 1997, **428**, 33–38.
7. C. Amatore, J. M. Saveant, D. Tessier, Charge transfer at partially blocked surfaces: A model for the case of microscopic active and inactive sites, *J. Electroanal. Chem*., 1983, **147**, 39-51.
